# Comparison of different vasodilators, endothelin antagonist, PDE5 inhibitior and sGC stimulators, in an animal model of secondary pulmonary hypertension: effects on “desaturation”

**DOI:** 10.1186/1471-2210-11-S1-P5

**Published:** 2011-08-01

**Authors:** Eva Maria Becker, Johannes-Peter Stasch, Martin Bechem, Hubert Truebel

**Affiliations:** 1 Bayer Schering Pharma AG, Cardiovascular Research, Wuppertal, Germany

## Background

Treatment options approved for pulmonary arterial hypertension (PAH) failed in secondary forms of pulmonary hypertension (PH) often related due to decrease in oxygenation. Therefore we established an animal model to evaluate different vasodilator mechanisms under experimental conditions of heterogeneous lung ventilation in respect to their “desaturation-potential”.

## Methods

Single-lung ventilation (right sided) was induced in 7-wk old minipigs (4-5 kg BW). In each animal 5 repetitive cycles of 10 min. left lobe blockade were followed by 30 min. bilateral ventilation. Hemodynamics (e.g. mean pulmonary artery pressure (mPAP), blood pressure (BP)) and arterial hemoglobin saturation (SaO2) were monitored continuously. We compared 5 different groups (n=6 each): vehicle control group, the endothelin antagonist bosentan (0.3, 1, 3, 10 mg/kg i.v.), the PDE5 inhibitor sildenafil (3, 10, 30, 100 µg/kg i.v.), and the sGC stimulators BAY 41-8543 (1, 3, 10, 30 µg/kg i.v.) and riociguat (1, 3, 10, 30 µg/kg i.v.). The vasodilator doses were chosen to achieve equal BP reduction. Cumulative doses of vasoactive compounds were applied before successive unilateral ventilation and effects on desaturation (area under the SaO2 curve, AUCSaO2) and maximal hypoxic mPAP were compared to vehicle conditions in each animal.

## Results

**Figure 1:**
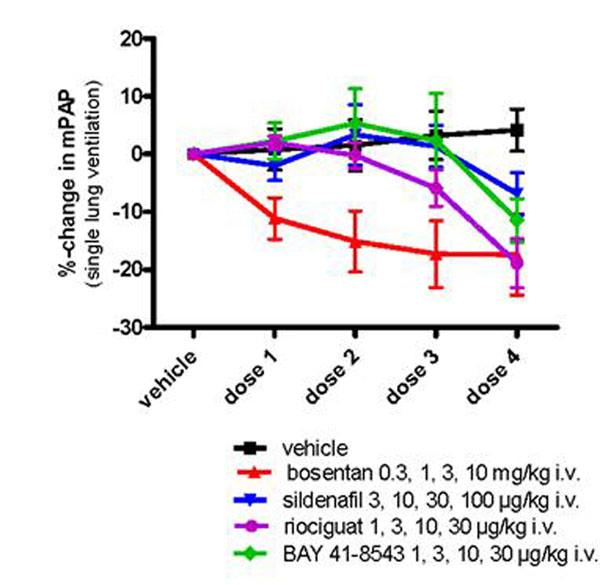


**Figure 2:**
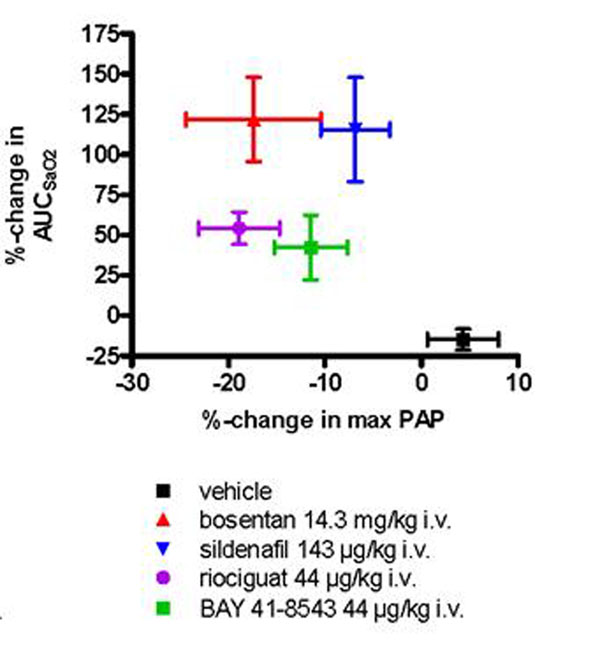


Single-lung ventilation resulted in transient increases in mPAP and desaturation (=increase in AUC_SaO2_). The vasodilators were compared in respect to their ability to decrease maximal hypoxic mPAP (positive treatment effect) and AUC_SaO2_ (unwanted desaturation effect). In contrast to vehicle treated control animals all vasodilators lead to a dose-dependent decrease in hypoxic mPAP (Figure 1) and an increase in AUC_SaO2_ during successive unilateral ventilation. Maximal changes in hypoxic mPAP associated with maximal increases in AUC_SaO2_ are shown in Figure 2 (mean ± SEM (n=6)).

## Conclusion

Beside the equal effects on BP, the studied compounds reduced hypoxic mPAP to a different extent with the strongest effect seen with the sGC stimulators (riociguat and BAY 41-8543) and bosentan. Furthermore, in comparison to bosentan and sildenafil the sGC stimulators were less likely to cause an unwanted decrease in SaO_2_. Explorations of these findings in patients with secondary PH might be warranted.

